# Association of Three Novel Inflammatory Markers: Lymphocyte to HDL‐C Ratio, High‐Sensitivity C‐Reactive Protein to HDL‐C Ratio and High‐Sensitivity C‐Reactive Protein to Lymphocyte Ratio With Metabolic Syndrome

**DOI:** 10.1002/edm2.479

**Published:** 2024-04-08

**Authors:** Rana Kolahi Ahari, Nazanin Akbari, Negin Babaeepoor, Zahra Fallahi, Sara Saffar Soflaei, Gordon Ferns, Mahmoud Ebrahimi, Mohsen Moohebati, Habibollah Esmaily, Majid Ghayour‐Mobarhan

**Affiliations:** ^1^ International UNESCO Center for Health‐Related Basic Sciences and Human Nutrition Mashhad University of Medical Sciences Mashad Iran; ^2^ Applied Biomedical Research Center Mashhad University of Medical Sciences Mashad Iran; ^3^ School of Nursing and Midwifery Mashhad University of Medical Sciences Mashad Iran; ^4^ Metabolic Syndrome Research Center Mashhad University of Medical Sciences Mashad Iran; ^5^ Division of Medical Education Brighton and Sussex Medical School Brighton UK; ^6^ Faculty of Medicine, Vascular and Endovascular Research Center Mashhad University of Medical Sciences Mashad Iran; ^7^ Department of Cardiology, Faculty of Medicine Mashhad University of Medical Sciences Mashad Iran; ^8^ Department of Biostatistics, School of Health, Social Determinants of Health Research Center Mashhad University of Medical Sciences Mashad Iran

**Keywords:** high‐sensitivity C‐reactive protein to HDL‐C ratio, high‐sensitivity C‐reactive protein to lymphocyte ratio, lymphocyte to HDL‐C ratio, metabolic syndrome

## Abstract

**Objective:**

We aimed to compare the association of three novel inflammatory indicators with metabolic syndrome (MetS) among Mashhad stroke and heart atherosclerotic disorder (MASHAD) cohort participants.

**Methods:**

According to the International Diabetes Federation (IDF) criteria, the cohort participants were divided into the MetS(+) and MetS(−) groups. The lymphocyte to high‐density lipoprotein cholesterol (HDL‐C) ratio (LHR), high‐sensitivity C‐reactive protein (hs‐CRP) to HDL‐C ratio (HCHR) and hs‐CRP to lymphocyte ratio (HCLR) were calculated and were compared between the groups. Binary logistic regression (LR) analysis was performed to find the association of the indices with the presence of MetS among men and women. Receiver‐operating characteristic (ROC) curve analysis was used to establish cut‐off values in predicting MetS for men and women. *p*‐Values <0.05 were considered as statistically significant.

**Results:**

Among a total of 8890 participants (5500 MetS(−) and 3390 MetS(+)), LHR, HCHR and HCLR were significantly higher in the MetS(+) group than in MetS(−) group (*p* < 0.001). In LR analysis, after adjusting for multiple cofounders, LHR remained an independent factor for the presence of MetS among men (OR: 1.254; 95% CI: 1.202–1.308; *p* < 0.001) and women (OR: 1.393; 95% CI: 1.340–1.448; *p* < 0.001). HCHR also remained an independent factor for the presence of MetS only in women (OR: 1.058; 95% CI: 1.043–1.073; *p* < 0.001). ROC curve analysis showed that LHR had the higher AUC for predicting MetS in both men (AUC: 0.627; 95% CI: 0.611–0.643; *p* < 0.001) and women (AUC: 0.683; 95% CI: 0.670, 0.696; *p* < 0.001).

**Conclusion:**

This suggests that among both genders, the LHR as an inexpensive and easy‐to‐access marker has a better diagnostic performance and could be a promising alternative to the traditional expensive inflammatory markers such as hs‐CRP for the evaluation of inflammation in patients with MetS.

## Graphical abstract

The article is about the association of novel inflammatory markers with metabolic syndrome (MetS). we found a novel and easy‐to‐access inflammatory marker, which may be an alternative to the traditional and expensive markers such as high‐sensitivity C‐reactive protein for the evaluation of inflammation in patients with MetS.

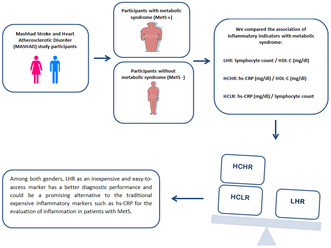

## 1 Introduction

Metabolic syndrome (MetS) is a cluster of cardiometabolic risk factors that increase the risk of cardiovascular diseases (CVDs), Type 2 diabetes mellitus (T2DM) and other health problems. These factors include impaired fasting glucose and insulin resistance, elevated blood pressure, high triglyceride levels, low high‐density lipoprotein cholesterol (HDL‐C) levels and central obesity [1, 2]. MetS is a highly common noncommunicable disease, and its prevalence and incidence increase globally [3]. Thus, the early identification of MetS is crucial to prevent cardiovascular and cerebrovascular complications [4]. It has been widely reported that MetS is a pro‐thrombotic and pro‐inflammatory condition that leads to low‐grade chronic inflammation and oxidative stress. Adipose tissue dysregulation in abdominal obesity plays a pivotal role in the promotion of inflammation in MetS [5, 6]. Circulating inflammatory biomarkers such as C‐reactive protein (CRP) and high‐sensitivity CRP (hs‐CRP) have also contributed to the inflammatory pathways of MetS [7]. An increasing body of evidence reported that changes in haematological parameters, including white blood cell (WBC), red blood cell (RBC) and platelet counts, as markers of pro‐inflammatory and pro‐thrombotic states, could be related to MetS and its components [8]. These findings about lymphocyte counts are inconsistent. Although some studies revealed that patients with MetS had decreased levels of lymphocyte counts, other investigations reported that increased levels of lymphocyte counts contributed to the pathogenesis of MetS.

On the contrary, HDL‐C as a component of MetS is a cardioprotective marker with contribution in the reverse cholesterol transport pathway and anti‐inflammatory, antioxidant and antithrombotic properties [9, 10]. However, in addition to the HDL‐C concentration, its function is also one of the factors influencing its anti‐inflammatory properties [11, 12].

In this study, we aimed to compare the association of three markers derived from lymphocyte counts, hs‐CRP and HDL‐C as novel and easy‐to‐assess inflammatory indicators with the presence of MetS among Iranian adult population and find whether they could use as an alternative marker of the conventional and expensive markers in showing the inflammatory state of MetS in clinical practice.

## 2 Materials and Methods

### 2.1 Study Design and Patient Population

This study was a secondary analysis of the Mashhad stroke and heart atherosclerotic disorder (MASHAD) study, which was a 10‐year prospective study in Mashhad City, northeast of Iran [13]. The MASHAD cohort study aimed to identify the risk and incidence of cardiovascular events among individuals aged between 35 and 65 years. The methodology and sampling details have been published elsewhere [13]. All participants included in the study were informed about the study, and the written informed consent was obtained before inclusion. The study was approved by the Ethics Committee of Mashhad University of Medical Sciences (MUMS), with the approval number of IR.MUMS.REC.1386.250.

**FIGURE 1:**
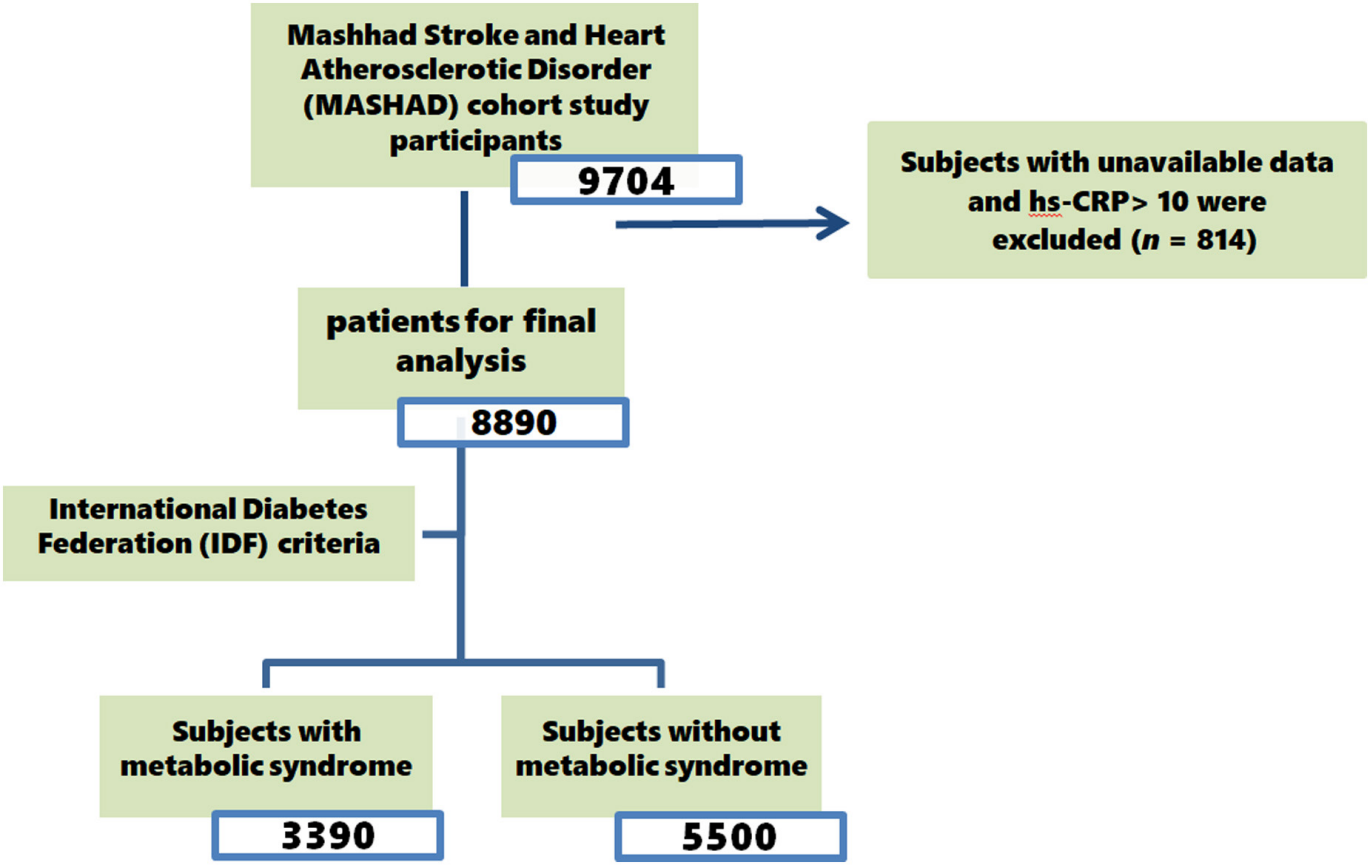
Flowchart of current study.

At the initial phase, subjects with co‐morbidities such as CVDs, malignancies and chronic kidney disease were excluded from the study. For the present study, we also excluded subjects with unavailable biochemical data and with hs‐CRP >10 mg/dL in order to reduce the possible risk of any infection or acute inflammation. The remaining population was classified as subjects with MetS (MetS(+)) and subjects without MetS (MetS(−)). The flowchart of the present study design is outlined in Figure 1.

### 2.2 The Exposure and Outcome Variable Definition

In present study, MetS was defined on the basis of the International Diabetes Federation (IDF) [14]. According to the IDF definition, someone has MetS if she or he has central obesity (≥94 cm for men and ≥80 cm for women) plus two or more of the following four factors:

1. raised TG concentration: ≥150 mg/dL (1.7 mmol/L) or receiving specific treatment for this lipid abnormality;
2. reduced HDL‐C concentration: <1.03 mmol/L (40 mg/dL) in males and <1.29 mmol/L (50 mg/dL) in females or receiving specific treatment for this lipid abnormality;
3. raised FBG concentration: ≥100 mg/dL (5.6 mmol/L) or previously diagnosed with T2DM;
4. raised blood pressure: systolic blood pressure (SBP) ≥130 mmHg or diastolic blood pressure (DBP) ≥85 mmHg or receiving specific treatment for previously diagnosed hypertension (HTN).

The inflammatory markers were calculated using the following formula [15, 16, 17]:

$$\mathrm{LHR}:\text{lymphocyte count}/\mathrm{HDL}‐C\;\left(\mathrm{mg}/\mathrm{dL}\right)$$

$$\text{HCHR}:\mathrm{hs}‐\mathrm{CRP}\;\left(\mathrm{mg}/\mathrm{dL}\right)/\mathrm{HDL}‐C\;\left(\mathrm{mg}/\mathrm{dL}\right)$$

$$\text{HCLR}:\mathrm{hs}‐\mathrm{CRP}\;\left(\mathrm{mg}/\mathrm{dL}\right)/\text{lymphocyte count}$$

### 2.3 Data Collection

After obtaining the informed consent forms, the socio‐demographic characteristics such as age, sex and smoking status were obtained. Height was measured by a standard wall height meter, and weight was measured by an analogue scale. Waist circumference (WC) was measured with a nonstretchable fibre measuring tape. The body mass index (BMI) was calculated as the weight (in kilogram) divided by the square of the height (meter). The blood pressure of the participants was taken from the right arm and repeated after 10 and 20 min (a total of three times). The mean of the three measurements was considered as the final blood pressure for each participant. Biochemical parameters including FBG, lipid profiles, hs‐CRP and haematologic data were taken by a 20‐mL blood sample between 8 and 10 AM through venipuncture of an antecubital vein after 14 h of overnight fasting and in a sitting position. BT3000 biochemical analyser was used for the determination of biochemical parameter levels.

### 2.4 Statistical Analysis

Continuous variables were expressed as mean ± standard deviation (SD), and categorical variables were expressed as number with percentage. The chi‐squared test was used for comparisons between two groups of categorical variables; the independent *t*‐test was used for continuous variables. Binary logistic regression (LR) analysis was used to find the association of the inflammatory markers with the presence of MetS among both genders in crude and adjusted models. Receiver‐operating characteristic (ROC) curve analysis was performed using the MedCalc statistical free trial software in order to find the predictive values of the inflammatory markers for MetS and cut‐off determination. All statistical analysis was performed using SPSS version 26.0 (IBM Corp., Armonk, NY). Two‐tailed *p*‐values < 0.05 were considered as statistically significant.

## 3 Results

### 3.1 Baseline Characteristics of the Study Population

As shown in Table 1, compared with the MetS(−) group, the MetS(+) group tended to be significantly older, more diabetic and hypertensive, with higher levels of BMI and larger WC (all *p* < 0.001). FBG, cholesterol, TG, LDL‐C, hs‐CPR and uric acid were also significantly higher in the MetS(+) group (all *p* < 0.001). Physical activity level (PAL) and HDL‐C were significantly lower (both *p* < 0.001) in the MetS(+) group than in the MetS(−) group. However, smoking status did not differ between the two groups (*p* = 0.317). In terms of haematological parameters, lymphocyte count (*p* < 0.001), neutrophil count (*p* = 0.001) and platelet count (*p* < 0.001) were significantly higher in the MetS(+) group but haemoglobin (*p* = 0.068) and haematocrit (*p* = 0.814) were not significantly different between the two groups. The MetS(+) group also has significantly higher levels of LHR, HCHR and HCLR (*p* < 0.001) than the MetS(−) group.

**TABLE 1:** Baseline and clinical characteristics of study population from Mashhad stroke and heart atherosclerotic disorder (MASHAD) study.

Characteristics	MetS(−)	MetS(+)	*p*‐Value
	(*n* = 5500, 61.90%)	(*n* = 3390, 38.10%)	
Sex			
Female	54.30%	68.00%	<0.001
Male	45.70%	32.00%	
Smoking status			
Smoker	21.50%	20.80%	0.317
Nonsmoker	69.00%	68.80%	
Ex‐smoker	9.50%	10.40%	
Diabetes			
Diabetic	6.70%	24.40%	<0.001
Nondiabetic	93.30%	75.60%	
HTN			
HTN+	16.90%	52.50%	<0.001
HTN−	83.10%	47.50%	
Age (year)	46.75 ± 8.16	50.01 ± 8.01	<0.001
BMI	26.93 ± 4.41	29.79 ± 4.13	<0.001
WC (cm)	91.07 ± 11.53	101.06 ± 9.72	<0.001
PAL	1.62 ± 0.29	1.54 ± 0.26	<0.001
SBP (mmHg)	115.86 ± 15.20	130.48 ± 19.24	<0.001
DBP (mmHg)	75.83 ± 9.93	84.05 ± 11.34	<0.001
Lymphocyte count (10^9^/L)	2.09 ± 1.71	2.27 ± 0.64	<0.001
Neutrophil count (10^9^/L)	3.20 ± 1.14	3.42 ± 3.85	0.001
Platelet count (10^9^/L)	225.75 ± 58.96	233.11 ± 61.52	<0.001
Haemoglobin (g/dL)	13.72 ± 1.59	13.79 ± 2.05	0.068
Haematocrit	41.22 ± 3.98	41.24 ± 3.82	0.814
Cholesterol (mg/dL)	185.54 ± 36.39	198.68 ± 40.82	<0.001
LDL‐C (mg/dL)	114.92 ± 32.86	118.38 ± 37.83	<0.001
TG (mg/dL)	111.34 ± 62.57	190.72 ± 109.14	<0.001
HDL‐C (mg/dL)	44.94 ± 10.37	39.48 ± 8.18	<0.001
FBG (mg/dL)	83.37 ± 25.99	104.68 ± 48.23	<0.001
Uric acid (mg/dL)	4.46 ± 1.37	4.88 ± 1.40	<0.001
Hs‐CRP (mg/dL)	1.99 ± 1.81	2.60 ± 2.04	<0.001
LHR	0.04 ± 0.02	0.06 ± 0.02	<0.001
HCHR	0.04 ± 0.04	0.06 ± 0.05	<0.001
HCLR	1.02 ± 0.99	1.23 ± 1.13	<0.001

Abbreviations: BMI, body mass index; DBP, diastolic blood pressure; FBG, fasting blood glucose; HCHR, hs‐CRP to HDL‐C ratio; HCLR, hs‐CRP to lymphocyte ratio; HDL, high‐density lipoprotein; hs‐CRP, high‐sensitive C‐reactive protein; HTN, hypertension; LDL, low‐density lipoprotein; LHR, lymphocyte to HDL‐C ratio; PAL, physical activity level; SBP, systolic blood pressure; TG, triglyceride; WC, waist circumference.

In order to determine the independent predictors for the presence of MetS, we used binary LR analysis for both men and women population. First, we used a crude (unadjusted) model and found that lymphocyte count (OR: 1.381; 95% CI: 1.240–1.539; *p* < 0.001), hs‐CRP (OR: 1.083; 95% CI: 1.043–1.124; *p* < 0.001), HCHR (OR: 1.055; 95% CI: 1.041–1.070; *p* < 0.001) and LHR (OR: 1.253; 95% CI: 1.213–1.259; *p* < 0.001) were associated with the presence of MetS among male population. Then, we adjusted the model with age, WC and PAL (Model 1). In this model, hs‐CRP lost its association and lymphocyte count (OR: 1.164; 95% CI: 1.026–1.320; *p* = 0.018), HCHR (OR: 1.026; 95% CI: 1.009–1.044; *p* = 0.003) and LHR (OR: 1.240; 95% CI: 1.191–1.291; *p* < 0.001) remained associated. We further adjusted the model with further possible cofounders such as uric acid and FBG (Model 2). In this model, only LHR (OR: 1.254; 95% CI: 1.202–1.308; *p* < 0.001) was an independent factor for the presence of MetS (Table 2). Among female population, we used the similar approach. In unadjusted model and Model 1, all variables were significantly associated with the presence of MetS. In Model 2, HCHR (OR: 1.058; 95% CI: 1.043–1.073; *p* < 0.001) and LHR (OR: 1.393; 95% CI: 1.340–1.448; *p* < 0.001) were independent factors for the presence of MetS (Table 3).

**TABLE 2:** Binary logistic regression analysis showing independent inflammatory factors of MetS in men.

Variable	Unadjusted model		Model 1		Model 2	
	OR (95% CI)	*p*‐Value	OR (95% CI)	*p*‐Value	OR (95% CI)	*p*‐Value
Lymphocyte	1.381 (1.240–1.539)	**<0.001**	1.164 (1.026–1.320)	**0.018** ^a^	—	—
hs‐CRP	1.083 (1.043–1.124)	**<0.001**	0.977 (0.930–1.027)	0.355	—	—
HCLR	1.029 (0.998–1.144)	0.057	0.920 (0.842–1.005)	0.065	0.930 (0.847–1.020)	0.125^b^
HCHR	1.055 (1.041–1.070)	**<0.001**	1.026 (1.009–1.044)	**0.003**	1.015 (0.996–1.034)	0.113^c^
LHR	1.253 (1.213–1.295)	**<0.001**	1.240 (1.191–1.291)	**<0.001**	1.254 (1.202–1.308)	**<0.001** ^d^

Abbreviations: HCHR, hs‐CRP to HDL‐C ratio; HCLR, hs‐CRP to lymphocyte ratio; hs‐CRP, high‐sensitive C‐reactive protein; LHR, lymphocyte to HDL‐C ratio.

*Note:* Bold values denote statistical significance at the *p* < 0.05 level.

^a^ Model 1: adjusted with age, WC, PAL.

^b^ Model 2: adjusted with variables in Model 1 + uric acid, FBG and HDL‐C.

^c^ Model 2: adjusted with variables in Model 1 + uric acid, FBG and lymphocyte.

^d^ Model 2: adjusted with variables in Model 1 + uric acid, FBG and hs‐CRP.

**TABLE 3:** Binary logistic regression analysis showing independent inflammatory factors of MetS in women.

Variable	Unadjusted model		Model 1		Model 2	
	OR (95% CI)	*p*‐Value	OR (95% CI)	*p*‐Value	OR (95% CI)	*p*‐Value
Lymphocyte	1.727 (1.569–1.899)	**<0.001**	1.521 (1.373–1.686)	**<0.001** ^a^	—	—
hs‐CRP	1.209 (1.175–1.245)	**<0.001**	1.124 (1.090–1.160)	**<0.001**	—	—
HCLR	1.255 (1.189–1.324)	**<0.001**	1.125 (1.062–1.192)	**<0.001**	1.051 (0.983–1.125)	0.143^b^
HCHR	1.119 (1.105–1.133)	**<0.001**	1.090 (1.076–1.105)	**<0.001**	1.058 (1.043–1.073)	**<0.001** ^c^
LHR	1.433 (1.384–1.483)	**<0.001**	1.424 (1.372–1.478)	**<0.001**	1.393 (1.340–1.448)	**<0.001** ^d^

Abbreviations: HCHR, hs‐CRP to HDL‐C ratio; HCLR, hs‐CRP to lymphocyte ratio; hs‐CRP, high‐sensitive C‐reactive protein; LHR, lymphocyte to HDL‐C ratio.

*Note:* Bold values denote statistical significance at the *p* < 0.05 level.

^a^ Model 1: adjusted with age, WC, PAL.

^b^ Model 2: adjusted with variables in Model 1 + uric acid, FBG and HDL‐C.

^c^ Model 2: adjusted with variables in Model 1 + uric acid, FBG and lymphocyte.

^d^ Model 2: adjusted with variables in Model 1 + uric acid, FBG and hs‐CRP.

**FIGURE 2:**
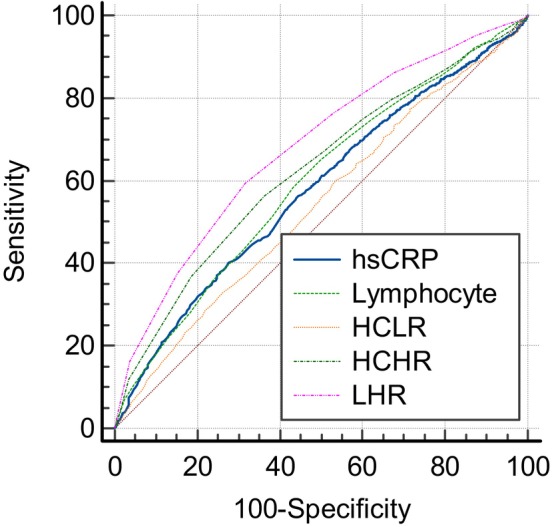
ROC curve analysis of inflammatory markers for predicting MetS in men.

**FIGURE 3:**
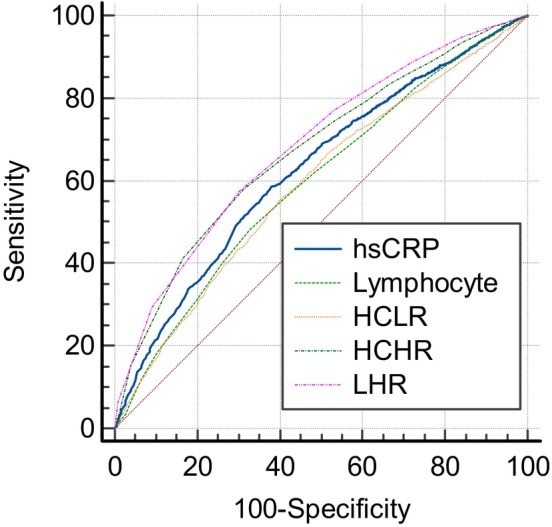
ROC curve analysis of inflammatory markers for predicting MetS in women.

We used the ROC curve analysis to find the predictive values of the inflammatory markers for MetS. The results revealed that among men, the cut‐off value for HCLR, HCHR and LHR was 0.45, 0.03 and 0.05, respectively. The area under curve (AUC) of HCLR, HCHR and LHR was 0.540 (*p* < 0.001; 95% CI: 0.524, 0.555), 0.627 (*p* < 0.001; 95% CI: 0.611, 0.643) and 0.679 (*p* < 0.001; 95% CI: 0.663, 0.695), respectively (Table 4). Also, among women, the cut‐off value for HCLR, HCHR and LHR was 0.76, 0.03 and 0.04, respectively. The AUC of HCLR, HCHR and LHR was 0.593 (*p* < 0.001; 95% CI: 0.579, 0.606), 0.673 (*p* < 0.001; 95% CI: 0.660, 0.685) and 0.683 (*p* < 0.001; 95% CI: 0.670, 0.696), respectively (Table 5). As shown in Figures 2 and 3, LHR had the highest AUC among inflammatory markers in both men and women.

**TABLE 4:** ROC curve analysis of inflammatory markers for predicting MetS in men.

Variable	Cut‐off value	Sensitivity (%)	Specificity (%)	AUC (95% CI)	*p*‐Value
Lymphocyte	2.10	56.61	58.60	0.594 (0.574, 0.613)	**<0.001**
hs‐CRP	1.09	40.53	71.96	0.577 (0.561, 0.594)	**<0.001**
HCLR	0.45	31.97	74.79	0.540 (0.524, 0.555)	**<0.001**
HCHR	0.03	56.72	63.84	0.627 (0.611, 0.643)	**<0.001**
LHR	0.05	59.54	68.13	0.679 (0.663, 0.695)	**<0.001**

Abbreviations: HCHR, hs‐CRP to HDL‐C ratio; HCLR, hs‐CRP to lymphocyte ratio; hs‐CRP, high‐sensitive C‐reactive protein; LHR, lymphocyte to HDL‐C ratio.

*Note:* Bold values denote statistical significance at the *p* < 0.05 level.

**TABLE 5:** ROC curve analysis of inflammatory markers for predicting MetS in women.

Variable	Cut‐off value	Sensitivity (%)	Specificity (%)	AUC (95% CI)	*p*‐Value
Lymphocyte	1.9	48.23	67.16	0.596 (0.583, 0.610)	**<0.001**
hs‐CRP	1.6	58.64	62.01	0.628 (0.615, 0.641)	**<0.001**
HCLR	0.76	55.16	60.36	0.593 (0.579, 0.606)	**<0.001**
HCHR	0.03	58.07	68.82	0.673 (0.660, 0.685)	**<0.001**
LHR	0.04	57.39	69.73	0.683 (0.670, 0.696)	**<0.001**

Abbreviations: HCHR, hs‐CRP to HDL‐C ratio; HCLR, hs‐CRP to lymphocyte ratio; hs‐CRP, high‐sensitive C‐reactive protein; LHR, lymphocyte to HDL‐C ratio.

*Note:* Bold values denote statistical significance at the *p* < 0.05 level.

## 4 Discussion

This study investigated the association between three less frequently used indicators of inflammation and the presence of MetS, and the main findings were as follows: LHR, HCHR and HCLR of patients with MetS were significantly higher than the patients without MetS. After adjusting with multiple cofactors, HCHR remained an independent factor for MetS in women and LHR remained an independent factor for MetS in men and women. ROC curve analysis showed that in both genders, the AUC of LHR was higher for the presence of MetS than of HCHR, HCLR, lymphocyte count and hs‐CRP alone. To the best of our knowledge, this study is the first clinical study to compare the association of LHR, HCHR and HCLR as novel and available inflammatory markers with MetS in both genders and among the large‐scale population.

The prevalence of MetS has been growing both in Iran and around the world, which is attributed to changes in lifestyle [18, 19, 20]. So far, many studies have investigated the relationship between different inflammatory markers and the presence of MetS in different countries. Song et al. [21] reported that hs‐CRP had a major role in the development of MetS in obese and nonobese women of Korea. Lai et al. [22] also demonstrated that hs‐CRP may be an important marker in the MetS pathophysiology, especially among women in Taiwan. Another study found that adiponectin levels had inverse association and that leptin and plasminogen activator inhibitor 1 levels had direct association with MetS among adolescents in Spain [23]. Kolahi et al. [24] reported serum uric acid to HDL‐C ratio as a novel inflammatory marker, which was correlated with the prevalence and severity of MetS among Iranian adult population. Also, many studies have demonstrated that alteration in haematological parameters is correlated with MetS [25, 26, 27, 28, 29, 30, 31, 32]. Although some studies have found that high lymphocyte counts were associated with inflammation and risk of MetS [33, 34, 35], others have reported that low lymphocyte count is associated with some inflammatory conditions [36, 37, 38].

HDL‐C, known as good cholesterol, is a cardioprotective factor, and the main effects are mostly attributed to its role in reverse cholesterol transport. HDL‐C also reduces oxidative stress in vascular smooth muscle cells and myocardium [39]. Interestingly, evidence suggests that along with the anti‐inflammatory, antioxidant and antithrombotic properties, HDL‐C is involved in the immune responses by the modulation of lipid raft components in immune cells [40]. Moreover, it has been reported that chronic inflammation can change the anti‐inflammatory function of HDL‐C to pro‐inflammatory function [11, 41].

In present study, we found that LHR, which is a combination of pro‐inflammatory and anti‐inflammatory markers, was an independent factor for MetS in both men and women (and higher in women than men) even after adjusting for multiple cofounders including age, WC, PAL, glucose level and uric acid level. Same as our results, Chen et al. [15] in a study on 852 Chinese adults found that LHR was an independent predictor for the presence of MetS after multiple adjustments (OR = 2.917, 95% CI: 2.110–4.033, *p* < 0.001). However, the study did not perform subgroup analysis on the basis of gender. They found that the AUC of LHR for predicting MetS was 0.694 (*p* < 0.001; 95% CI: 0.655–0.732), which is almost similar to ours (0.679 (*p* < 0.001; 95% CI: 0.663–0.695) for men and for women 0.683 (*p* < 0.001; 95% CI: 0.670–0.699)). Also, Yu et al. in a prospective study with 4.66 years of follow‐up found that LHR was an effective predictor of newly diagnosed MetS in the subjects of rural China. They also found a graded relationship between LHR and increasing in numbers of metabolic factors. Chen et al. found that LHR was associated with higher risk of MetS in both men and women. However, when additional adjustments were made, LHR remained associated in only women [25]. We found that LHR was associated with risk of MetS in both genders after full adjustments, which was stronger association rather than hs‐CRP. Thus, maybe it could be used as a promising alternative to hs‐CRP for the evaluation of inflammation in patients with MetS.

A possible explanation about the association between haematological parameters and MetS is related to insulin resistance. It has been widely reported that insulin and Insulin Growth Factors (IGF) I and II promote the RBC and WBC proliferation [42, 43, 44, 45]. Moreover, the low‐grade chronic inflammation related to MetS could increase the inflammatory cytokines including tumour necrosis factor (TNF)‐α and interleukin (IL)‐8 leading to leucocytosis [29, 46].

HCHR is another combination of pro‐inflammatory and anti‐inflammatory markers. Although the association between HCHR and conditions such as coronary artery disease (CAD) [16] and contrast‐induced acute kidney injury [47] has been discussed before, no clinical studies investigate the association between HCHR and MetS yet. We found that HCHR, independent of multiple factors, is associated with MetS in women. However, as the co‐founding factors for adjustment increased, HCHR lost its association with MetS in men.

HCLR as another novel inflammatory biomarker was significantly higher in the MetS(+) group than in the MetS(−) group in our study. However, in binary LR analysis, it was not an independent factor for MetS in men. Also, when the cofounding factors for adjustment increased, HCLR lost its association with MetS in women too. The predictive value of HCLR in the prognosis of hepatocellular carcinoma was also investigated before [17]. Liao et al. [17] found that the increased preoperative levels of HCLR were significantly associated with overall survival and progression‐free survival in patients with hepatocellular carcinoma after surgical resection. According to our findings, HCLR did not seem to have priority over hs‐CRP or lymphocyte alone in the evaluation of MetS.

Our study has some limitations, which need to be considered. As this study was a cross‐sectional study, the assessment of causality relationship of HCLR, HCHR and LHR with MetS was not possible. Also, the lack of assessment inflammatory cytokines and other inflammatory indicators may affect the results. Moreover, as this study was performed among the Iranian population, the results may not be generalised to other ethnicities. We suggest more longitudinal cohort studies in order to better find the clinical applications of LHR, HCHR and HCLR for the presence and incident of MetS.

## 5 Conclusion

This suggests that among both genders, the LHR as an inexpensive and easy‐to‐access marker has a better diagnostic performance and could be promising alternatives to the traditional and expensive inflammatory markers such as hs‐CRP for the evaluation of inflammation in subjects with MetS. More investigations are needed to determine the applications of LHR, HCHR and HCLR in other disorders.

## Author Contributions

**Rana Kolahi Ahari:** Conceptualization (equal); formal analysis (equal); methodology (equal); writing – original draft (equal); writing – review and editing (equal). **Nazanin Akbari:** Data curation (equal); writing – original draft (equal). **Negin Babaeepoor:** Data curation (equal). **Zahra Fallahi:** Data curation (equal). **Sara Saffar soflaei:** Conceptualization (equal); methodology (equal); writing – review and editing (equal). **Gordon Ferns:** Writing – review and editing (equal). **Mahmoud Ebrahimi:** Supervision (equal). **Mohsen Moohebati:** Supervision (equal). **Habibollah Esmaily:** Supervision (equal). **Majid Ghayour‐Mobarhan:** Conceptualization (equal); project administration (lead).

## Ethics Statement

The ethics approval of the present study was obtained from the Ethics Committee of MUMS (ethics approval code: 85134). All participants signed a written informed consent form before inclusion.

## Conflicts of Interest

The authors declare no conflicts of interest.

## Data Availability

The data sets used during the present study are available from the corresponding author on reasonable request.
